# Seasonal Diet Variation in Reintroduced Milu (*Elaphurus davidianus*) Revealed by DNA Macrobarcoding: Implications for Biodiversity Conservation

**DOI:** 10.3390/ani16162544

**Published:** 2026-08-14

**Authors:** Menglin Sun, Yifan Zhou, Hong Wu, Dapeng Zhao

**Affiliations:** Tianjin Key Laboratory of Conservation and Utilization of Animal Diversity, College of Life Sciences, Tianjin Normal University, Tianjin 300387, China; sml7652924@163.com (M.S.); m19139711931@163.com (Y.Z.)

**Keywords:** *Elaphurus davidianus*, seasonal dietary variation, DNA metabarcoding, foraging strategy, wetland conservation

## Abstract

Understanding seasonal dietary shifts in reintroduced herbivores is essential for conservation, yet detailed patterns for Milu (*Elaphurus davidianus*) in wetland habitats remain underexplored. This study applied high-throughput sequencing to fecal samples collected across four seasons in Tianjin Qilihai Wetland, identifying 43 plant species from 24 families. Significant seasonal differences were detected in relative read abundance. At the family level, Poaceae dominated spring, significantly exceeding all other seasons; Asteraceae and Cyperaceae were highest in summer, with Cyperaceae also surpassing winter and spring; Amaranthaceae peaked in autumn, higher than spring and summer; and Celastraceae prevailed in winter. At the species level, *Phragmites australis* was overwhelmingly consumed in spring; *Lactuca indica* and *Bolboschoenus planiculmis* peaked in summer; *Euonymus japonicus* dominated winter; and *Suaeda salsa* was highest in autumn, exceeding spring. These compositional shifts reveal a strategic transition from a specialized diet focused on high-quality resources in spring–summer to a broad-spectrum diet including woody plants, legumes, and cultivated species in autumn–winter. Overall, Milu exhibit substantial dietary plasticity, a key trait underpinning reintroduction success, and our findings provide empirical evidence to guide adaptive forage management in wetland reserves.

## Simple Summary

Seasonal dietary shifts in reintroduced Milu (*Elaphurus davidianus*) in wetland ecosystems remain poorly documented. We used high-throughput sequencing to analyze fecal samples from Tianjin Qilihai Wetland across four seasons, identifying 43 plant species. Diet composition varied significantly among seasons: spring was dominated by *Phragmites australis*; summer was dominate by forbs such as *Lactuca indica* and *Bolboschoenus planiculmis*; autumn showed increased use of Amaranthaceae and the protected *Glycine soja*; while winter relied heavily on cultivated *Euonymus japonicus*. These changes represent a strategic transition from a specialized high-quality resource diet in spring–summer to a broad-spectrum diet including woody and cultivated plants in autumn–winter, consistent with optimal foraging theory. Our results highlight Milu’s remarkable dietary plasticity, which supports reintroduction success, and provide practical guidance for managing food resources in wetland reserves.

## 1. Introduction

Research on wildlife diet is fundamental to understanding species’ survival strategies, ecological behavior, and energy flow within ecosystems [1,2,3,4,5,6] and is particularly critical for the conservation and management of endangered species [7,8]. Traditional dietary analysis methods, such as direct observation, food trace analysis, and microscopic histological analysis of feces, generally face challenges including low classification resolution, weak quantitative ability, high time consumption, and high subjectivity [9,10]. It may overestimate the proportion of some plant groups that are difficult to digest [11]. By contrast, DNA metabarcoding technology substantially improves both the detection rate and accuracy of diet composition assessments [12,13], and has been widely applied in dietary research in recent years [6,7,14,15,16,17]. Based on high-throughput sequencing of residual environmental DNA in fecal samples and matching sequences against barcode reference databases, this technique could achieve more accurate and reliable characterization of animal diet composition, offering unprecedented opportunities to comprehensively and precisely reveal the true dietary spectra of animals and their seasonal dynamics [15,18].

Currently, the accuracy of this technique is highly dependent on the completeness and reliability of the reference sequence database; for a specific study area, constructing a high-quality barcode reference library that covers the local potential food species is a fundamental prerequisite for overcoming the problems of missing sequences or mislabeled entries in public databases and for ensuring accurate and reliable identification [19,20]. For example, Littleford-Colquhoun et al. (2024) collected plant specimens directly from Yellowstone National Park to build a local reference library comprising 191 *trnL-P6* sequences targeting the diets of large herbivorous mammals, effectively resolving taxonomic ambiguities present in global databases and markedly improving the accuracy of dietary analysis [20]. Botha et al. (2023) established a local reference library covering *rbcL* and trnL markers for a semi-arid grassland in South Africa, which could accurately identify rare plant species in the ecosystem [21]. Kartzinel et al. (2015) used DNA metabarcoding in combination with a self-built reference library to reveal dietary niche partitioning among large herbivorous mammals in Africa [8]. The results showed that even among sympatric “grazers,” the specific grass species consumed differed significantly, providing new perspectives for understanding species coexistence mechanisms.

As one typical representative species in conservation biology, the Milu is the only species in the genus *Elaphurus* (Mammalia, Artiodactyla, Cervidae) and a wetland deer historically endemic to China. Owing to excessive hunting and habitat destruction, the Milu was driven to extinction in the wild in China by the late 19th century. The species was reintroduced to China in 1980S [22]. Its successful reintroduction represents a landmark achievement in wildlife conservation, and both its population recovery and habitat adaptation have since attracted considerable scientific attention [22]. Subsequently, in 2011, the Tianjin Qilihai Wetland received Milu individuals from Beijing. Systematically elucidating its diet composition and seasonal variation patterns can not only reveal its resource utilization strategy, but also help assess habitat quality and predict the impacts of environmental change, thereby providing critical ecological evidence on developing scientific population and habitat management plans. To date, several studies have provided data on the dietary composition of Milu in different habitats, but most have employed traditional fecal microhistological analysis [23] and stable isotope technology [24,25]. Only two studies have employed DNA metabarcoding to investigate its dietary characteristics [26,27], yet neither encompassed the complete spectrum of seasonal dietary shifts. Moreover, Lin et al. (2024) identified food items only to the plant genus level, which constrained fine-scale analysis of dietary composition [26]. Based on the background mentioned above, the present study is the first to integrate DNA metabarcoding with a self-constructed local reference sequence library to accurately characterize the full seasonal dietary composition of the Milu population in the Qilihai Wetland, Tianjin, thereby providing more comprehensive and in-depth empirical evidence for the conservation biology of this species.

## 2. Materials and Methods

### 2.1. Research Location and Sample Collection

**Figure 1:**
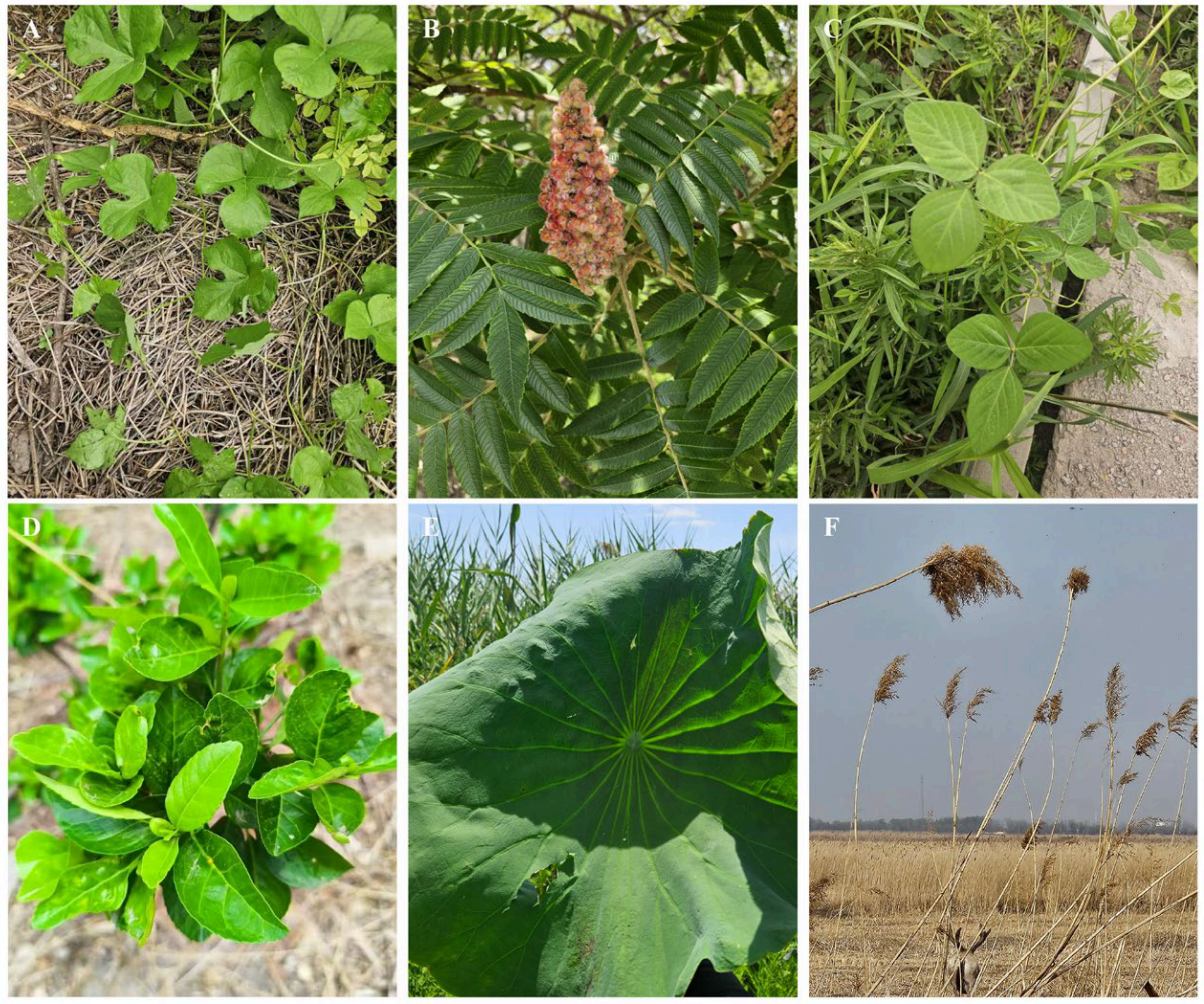
Representative images of selected plant samples ((**A**) *Ipomoea nil*, (**B**) *Rhus typhina*, (**C**) *Glycine soja*, (**D**) *Euonymus japonicus*, (**E**) *Nelumbo nucifera*, (**F**) *Phragmites australis*).

Fecal samples of the Milu were collected from Tianjin Qilihai Wetland, Tianjin City, China, from July 2024 to March 2025. The core area of the Tianjin Qilihai Wetland in Tianjin covers approximately 233.49 km^2^. The region has a warm temperate semi-arid and semi-humid monsoon climate. The main habitat types include extensive reed marshes, shallow water areas, and mudflats. To minimize the likelihood of repeated sampling of the same individual, fresh fecal pellet groups were collected only when the straight-line distance between groups exceeded 5 m, a threshold determined by preliminary behavioral observations of the Milu herd. In addition, pellet freshness, size, and shape were recorded and compared; pellet groups that were spatially close (<5 m) and morphologically similar were excluded to reduce potential duplicates. Sampling was further guided by real-time observation of the herd using binoculars to ensure collection from spatially distinct animal clusters. Fresh fecal samples were obtained using sterile forceps, and to reduce contamination, only the inner portion of the feces that had not been in contact with the ground or vegetation was transferred into sterile 50 mL microcentrifuge tubes for subsequent experiments. All fresh fecal samples were temporarily stored in a portable insulated cooler, transported to the laboratory as quickly as possible, and then stored at −80 °C in an ultra-low temperature freezer until DNA extraction. The Qilaihai Wetland in Tianjin is located in the warm temperate, semi-humid monsoon climate zone, with distinct four seasons. March to May is spring, June to August is summer, September to November is autumn, and December to February is winter [28]. The fecal samples were divided into four seasonal groups: spring, summer, autumn, and winter (sample group names and identifiers were presented in Table 1). In addition, 93 plant species from the study area were collected to construct a local plant sequence database. Representative images of some collected plant samples are shown in Figure 1.

### 2.2. DNA Extraction and Establishment of Local Database

The plant chloroplast *rbcL* gene fragment has been widely used in dietary studies of herbivores [6,7,29,30]. In this study, DNA of the 93 collected plant samples was extracted using the CTAB method, and the primer pair Z1aF (5′-ATGTCACCACCAACAGAGACTAAAGC-3′) and hp2R (5′-CGTCCTTTGTAACGATCAAG-3′) (Sangon Biotech, Beijing, China) was used to amplify the plant chloroplast *rbcL* gene fragment for constructing a local plant DNA sequence database. In addition to the experimental plant samples, two blank samples were included as negative controls in each batch of extraction.

Total DNA of each fecal sample was extracted using the QIAamp Fast DNA Stool Mini Kit (QIAGEN, Hilden, Germany). To ensure that no contamination occurred, two blank samples were likewise included as negative controls in each batch of extraction. The concentration and quality of the extracted DNA were measured using a NanoDrop 2000 spectrophotometer (Thermo Fisher Scientific, Wilmington, DE, USA). The chloroplast *rbcL* primers Z1aF (5′-ATGTCACCACCAACAGAGACTAAAGC-3′) and hp2R (5′-CGTCCTTTGTAACGATCAAG-3′) were used to amplify plant DNA from the Milu fecal samples, and two blank controls were set in each amplification run. The PCR amplification system contained 4.0 μL of 5 × FastPfu Buffer (TransGen Biotech, Beijing, China), 2.0 μL of 2.5 mmol/L dNTPs, 0.8 μL of each forward and reverse primer (1 μmol/L), 0.4 μL of FastPfu polymerase (TransGen Biotech, Beijing, China), 10 ng of template DNA, and was made up to 20 μL with ddH_2_O. The amplification products were examined by 1% agarose gel electrophoresis, and no bands were observed in the blank samples. After detection, the PCR products were purified and subsequently subjected to high-throughput sequencing on an Illumina MiSeq platform by Shanghai Meiji Biomedical Technology Co., Ltd. (Shanghai, China).

### 2.3. Data Analysis

Raw paired-end reads were processed using Fastp (version 0.23.4) and then the clean reads from each sample were merged using FLASH (version 1.2.11). OTU sequences were aligned against the established local plant reference database for taxonomic assignment, using a sequence identity and coverage threshold of 98% and an E-value cutoff of 1 × 10^−5^. Community bar plots were used to display the dietary composition at the family and species levels across different groups. For alpha diversity analysis, the Ace, Simpson, Shannon, Chao1 and Pielou’s evenness indices were calculated. The Kruskal–Wallis test was applied to assess the significance of differences in alpha diversity among groups, and post-hoc comparisons were performed using the Tukey–Kramer test. Beta diversity distance matrices were computed using QIIME (version 1.91). Based on these matrices, principal coordinates analysis (PCoA) and ANOSIM were conducted. Ordination plots were created in R (version 3.3.1). The Kruskal–Wallis H test was employed to examine whether major dietary components at the family and species levels differed significantly among seasons. The five plant taxa with the highest mean relative abundances among those exhibiting significant differences were identified, and post-hoc comparisons were carried out using the Tukey–Kramer test.

In the statistical analysis results of this study, differences significant at 0.01 ≤ *p* < 0.05 were defined as significant and indicated with “*” in the relevant graphs. Differences significant at 0.001 ≤ *p* < 0.01 were defined as extremely significant and indicated with “**”. Differences significant at *p* ≤ 0.001 were defined as super-significant and indicated with “***”.

## 3. Results

### 3.1. Main Dietary Composition

After high-throughput sequencing and denoising of 34 samples from four seasonal groups, a total of 813,892 valid sequences were obtained (Table S3), comprising 43 species, 42 genera, 24 families, and 15 orders of plants. The summer group’s diet included 29 species, 28 genera, 17 families, and 11 orders. The autumn group’s diet included 34 species, 33 genera, 17 families, and 12 orders. The winter group’s diet included 32 species, 31 genera, 17 families, and 12 orders. The spring group’s diet included 32 species, 31 genera, 18 families, and 12 orders (Table S5). The Shannon curve flattened out after climbing to a certain point, which indicates that our sequencing depth was sufficient to capture the full species diversity in the samples (Figure S1).

**Figure 2:**
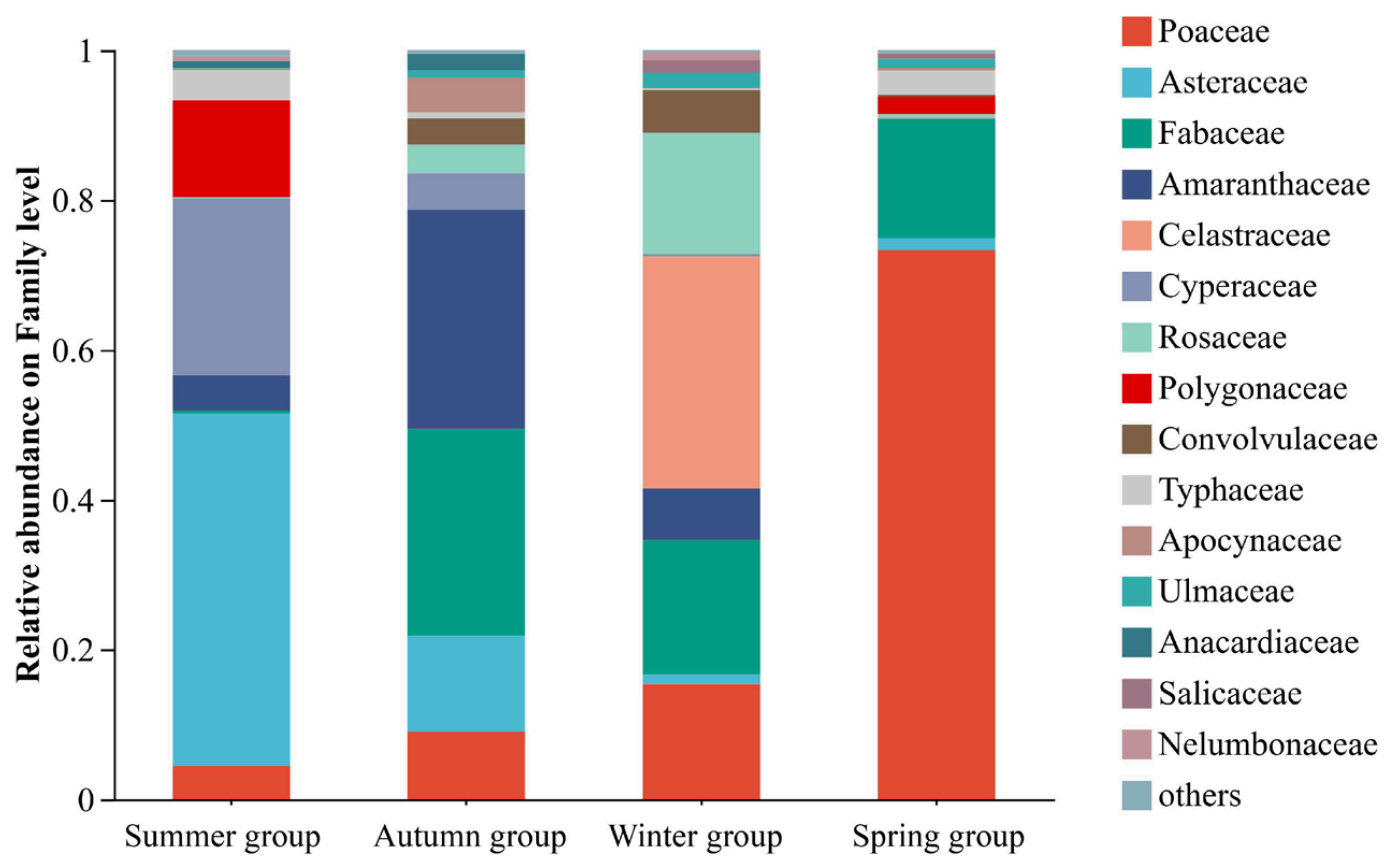
Plant composition at the family level.

At the family level, the top five families in the summer group’s diet included Asteraceae (47.42%), Cyperaceae (24.42%), Polygonaceae (13.55%), Poaceae (4.53%), and Typhaceae (4.00%). In the autumn group, the top five families were Amaranthaceae (29.12%), Fabaceae (27.67%), Asteraceae (12.74%), Poaceae (9.06%), and Cyperaceae (4.93%). In the winter group, the top five families were Celastraceae (31.05%), Fabaceae (17.79%), Rosaceae (17.13%), Poaceae (15.45%), and Amaranthaceae (7.00%). In the spring group, the top five families were Poaceae (73.39%), Fabaceae (15.97%), Typhaceae (3.23%), Polygonaceae (2.36%), and Asteraceae (1.53%) (Figure 2). Among these, Poaceae was the common dominant plant family consumed in all seasons (Table S2).

**Figure 3:**
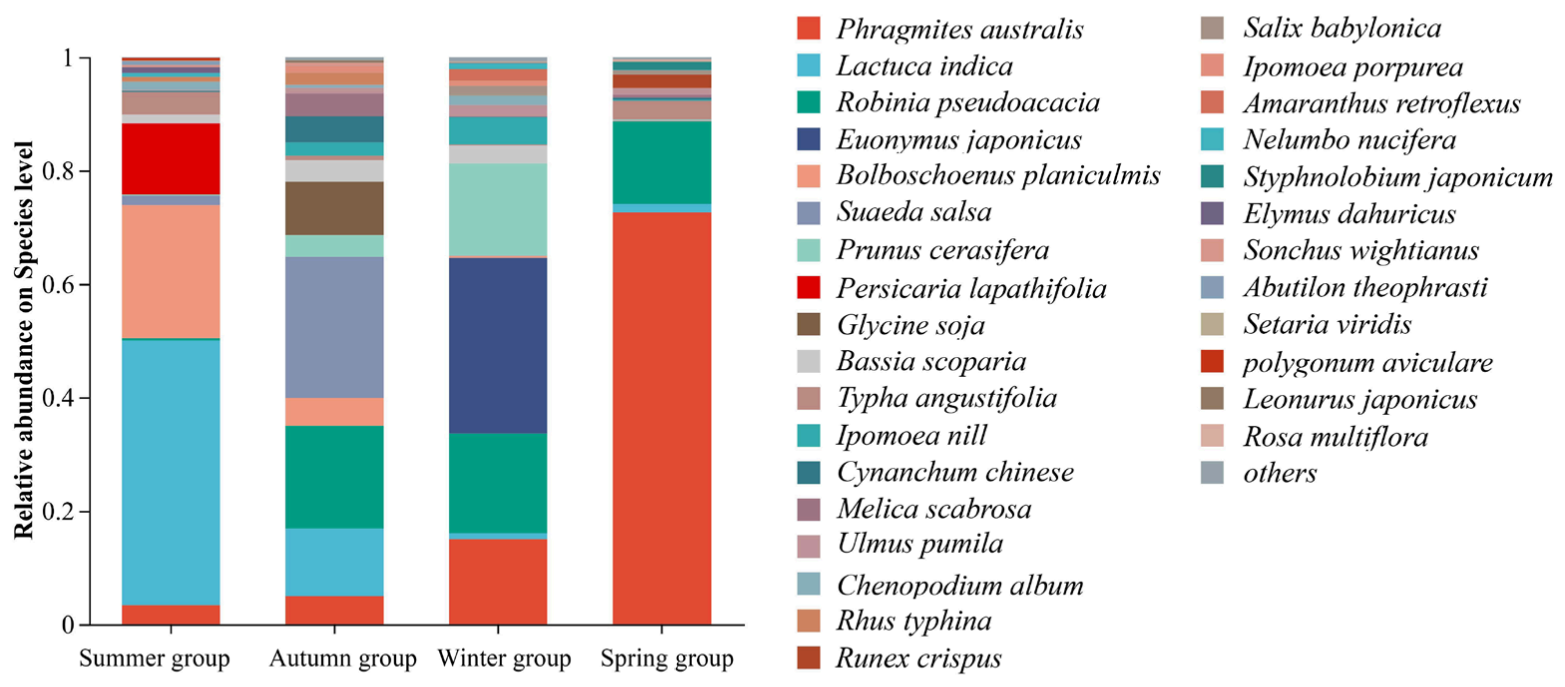
Plant composition at the species level.

At the species level, the top five plants in the summer group’s diet were *Lactuca indica* (47.00%), *Bolboschoenus planiculmis* (24.42%), *Persicaria lapathifolia* (13.08%), *Typha angustifolia* (4.00%), and *Phragmites australis* (3.49%). In the autumn group, the top five plants were *Suaeda salsa* (24.89%), *Robinia pseudoacacia* (18.09%), *Lactuca indica* (11.98%), *Glycine soja* (9.49%), and *Phragmites australis* (4.97%). In the winter group, the top five plants were *Euonymus japonicus* (31.06%), *Robinia pseudoacacia* (17.54%), *Prunus cerasifera* (17.13%), *Phragmites australis* (15.17%), and *Ipomoea nil* (4.80%). In the spring group, the top five plants were *Phragmites australis* (72.79%), *Robinia pseudoacacia* (14.55%), *Typha angustifolia* (3.23%), *Lactuca indica* (1.49%), and *Ulmus pumila* (1.13%) (Figure 3). Among these, *Phragmites australis* was the common dominant plant species consumed among all seasons (Table S1).

### 3.2. Seasonal Comparisons

**Figure 4:**
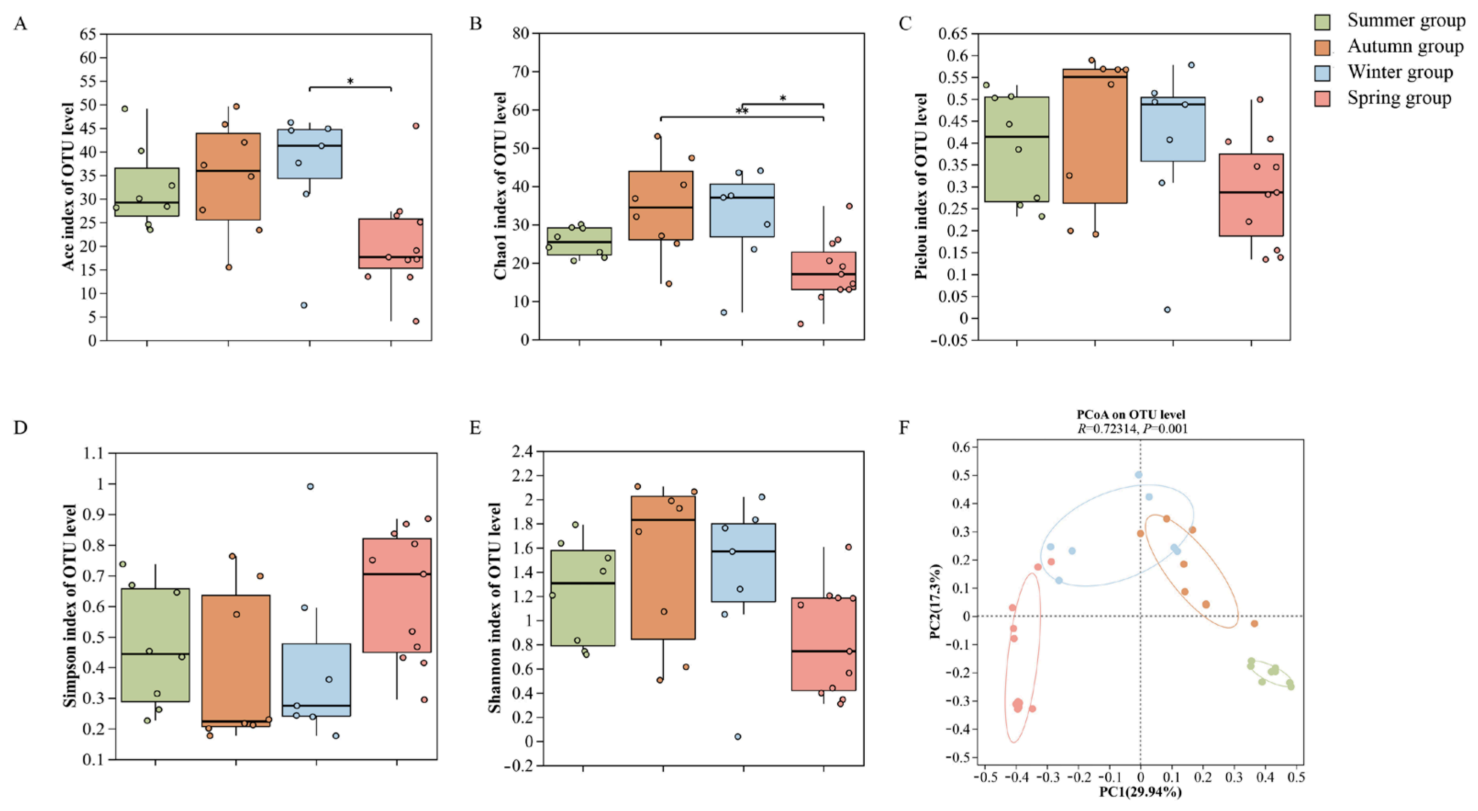
Alpha diversity and Beta diversity analyses ((**A**) Ace index, (**B**) Chao1 index, (**C**) Pielou index, (**D**) Simpson index, (**E**) Shannon index, (**F**) PCoA analysis). *: 0.01 ≤ *p* < 0.05, **: 0.001 ≤ *p* < 0.01.

Alpha diversity analysis showed that there were no significant differences in the Pielou evenness index, Shannon index, or Simpson index among the four seasonal groups (Table S4). The Ace and Chao1 indices of the autumn group were significantly higher than those of the spring group, indicating that the dietary richness of Milu in autumn was significantly higher than that in spring (Figure 4). Beta diversity analysis revealed that samples within each seasonal group clustered together, while samples among groups were clearly distinguishable. The ANOSIM test further confirmed significant differences among groups (*p* = 0.001) (Figure 4).

**Figure 5:**
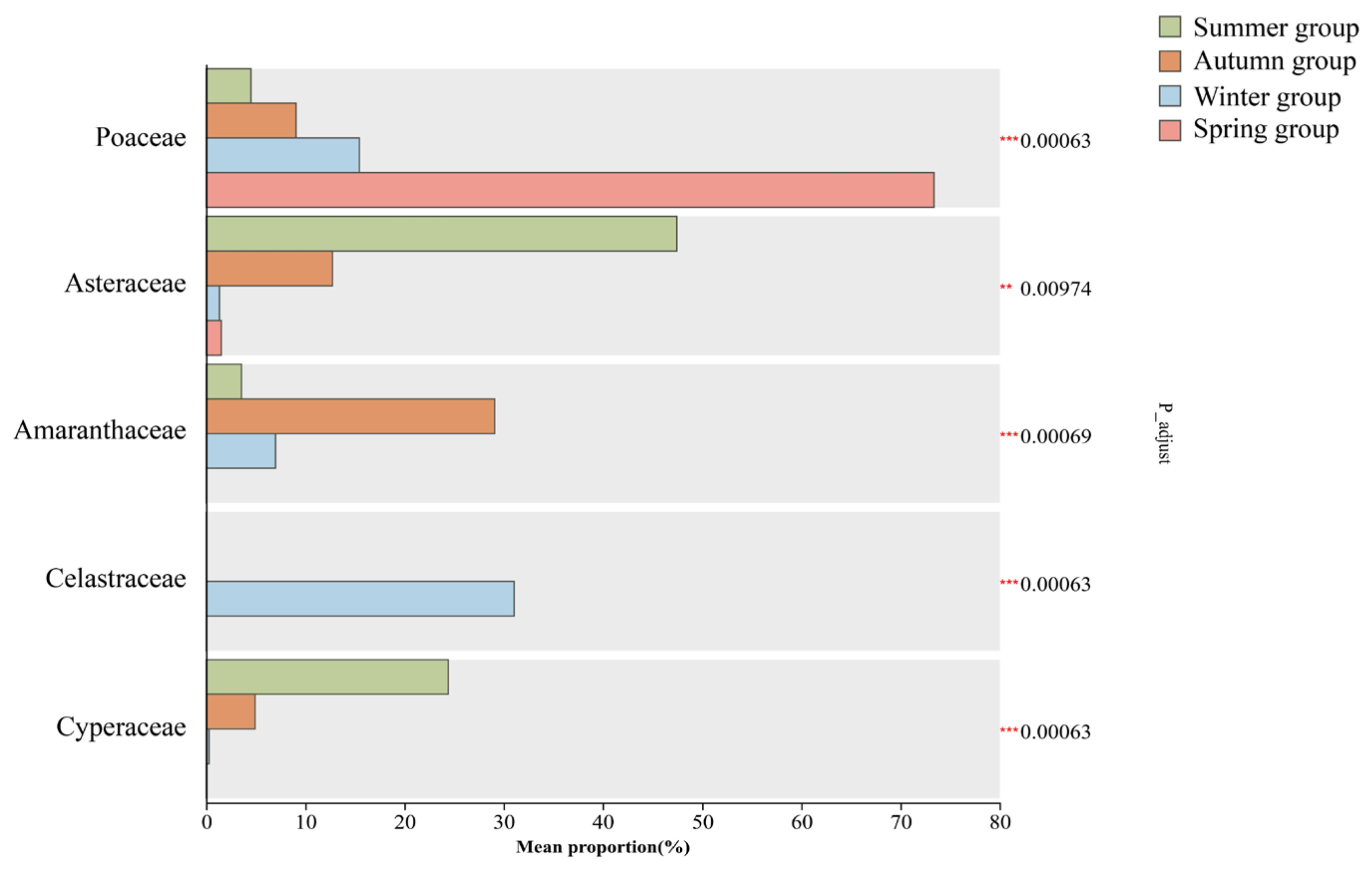
Kruskal–Wallis H test showing significant differences in plant families consumed by Milu deer across four seasons (Top 5 families by mean abundance). **: 0.001 ≤ *p* < 0.01, ***: *p* ≤ 0.001.

**Figure 6:**
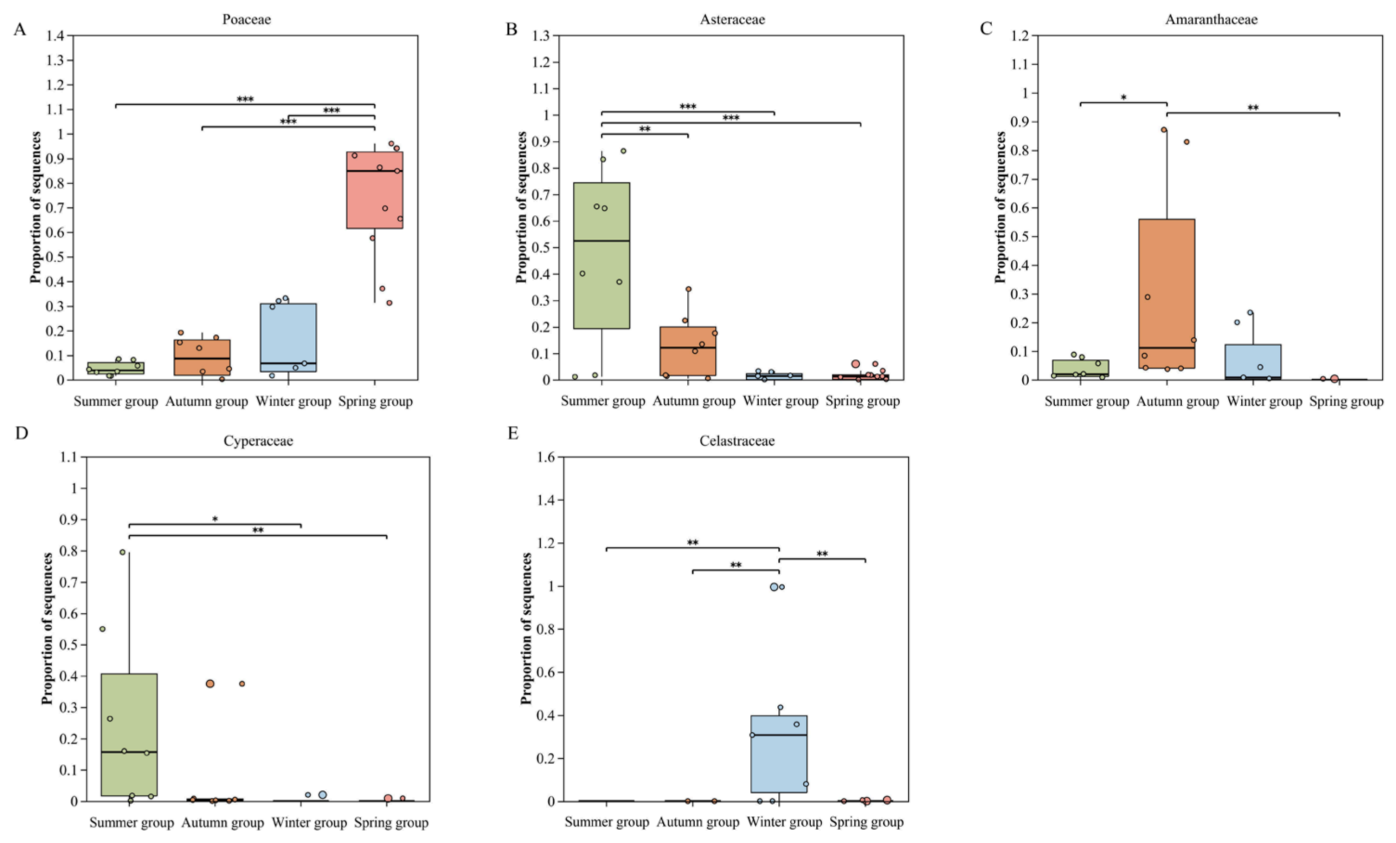
Pairwise comparisons of individual plant families showing significant differences between groups at the family level ((**A**) Poaceae, (**B**) Asteraceae, (**C**) Amaranthaceae, (**D**) Cyperaceae, (**E**) Celastraceae). *: 0.01 ≤ *p* < 0.05, **: 0.001 ≤ *p* < 0.01, ***: *p* ≤ 0.001.

It was found that, at the family level, the top five families with the highest mean differences in relative read abundance across four seasons were Poaceae, Asteraceae, Amaranthaceae, Celastraceae, and Cyperaceae (Figure 5). Specifically, the proportion of Poaceae in spring was significantly higher than that in the other three seasons; the proportion of Asteraceae in summer was significantly higher than that in the other three seasons, and the proportion of Cyperaceae in summer was significantly higher than that in winter and spring; the proportion of Amaranthaceae in autumn was significantly higher than that in spring and summer; and the proportion of Celastraceae in winter was significantly higher than that in the other three seasons (Figure 6).

**Figure 7:**
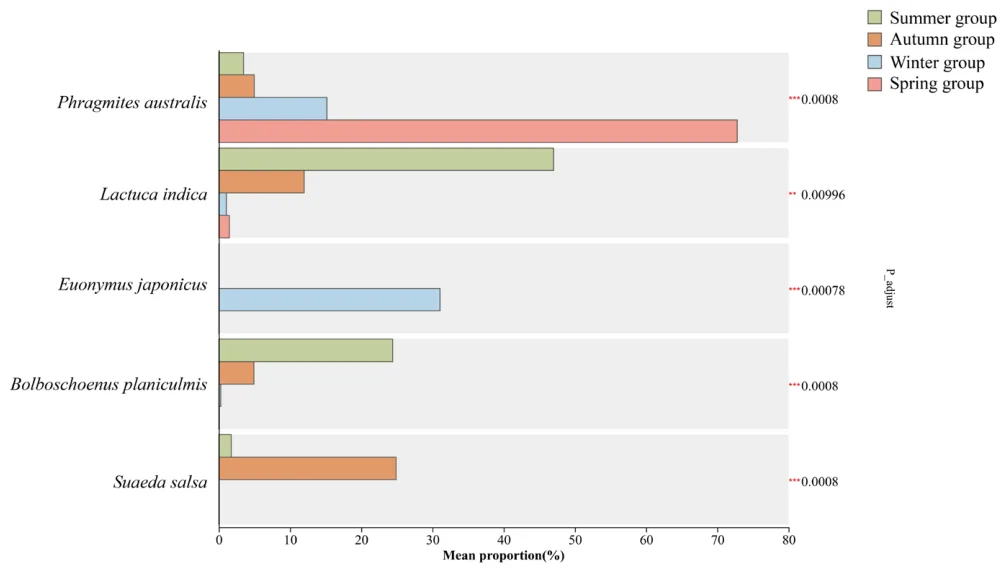
Kruskal–Wallis H test showing significant differences in plant species consumed by Milu deer across four seasons (Top 5 species by mean abundance). **: 0.001 ≤ *p* < 0.01, ***: *p* ≤ 0.001.

**Figure 8:**
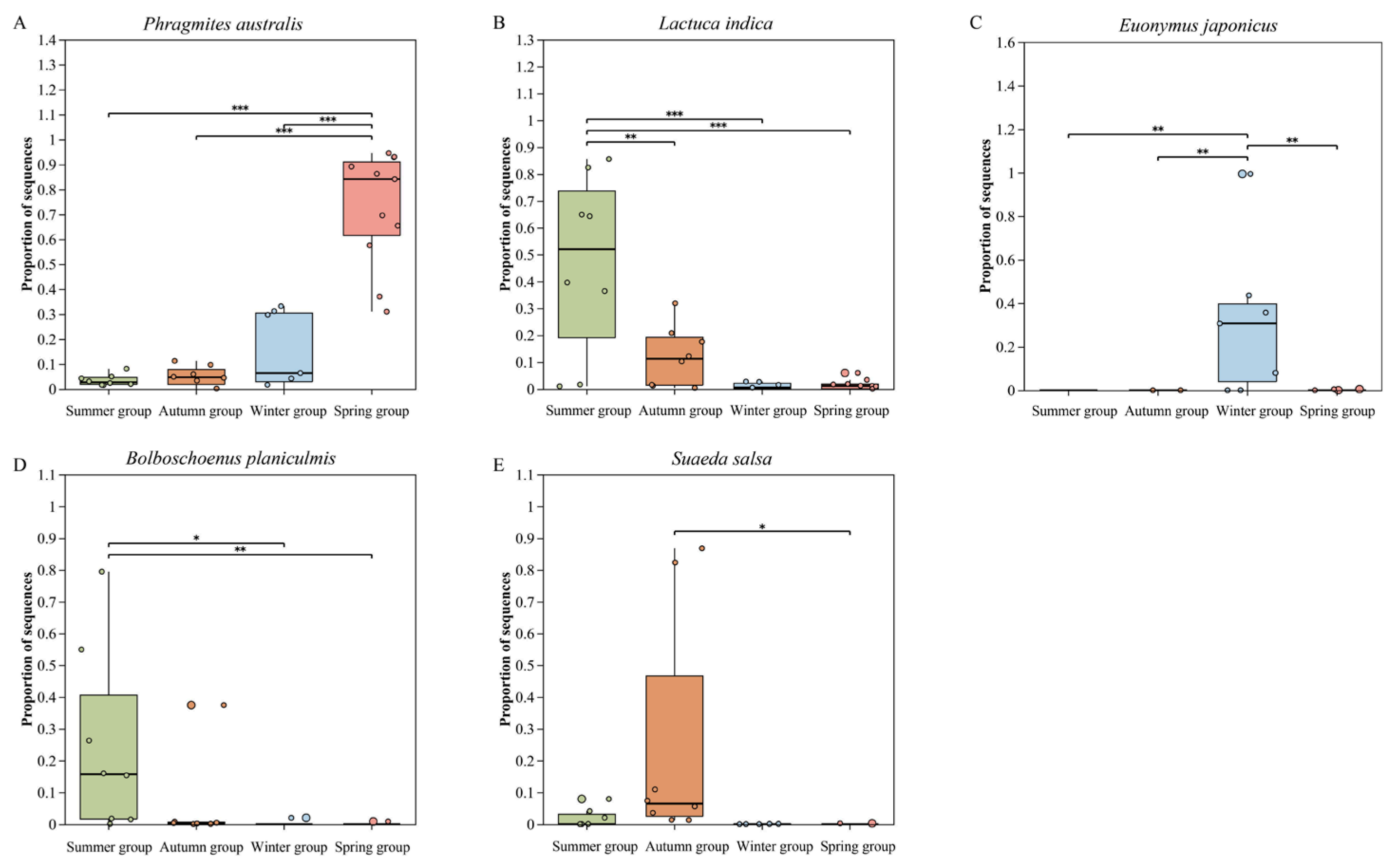
Pairwise comparisons of individual plant species showing significant differences between groups at the species level ((**A**) *Phragmites australis*, (**B**) *Lactuca indica*, (**C**) *Euonymus japonicus*, (**D**) *Bolboschoenus planiculmis*, (**E**) *Suaeda salsa*). *: 0.01 ≤ *p* < 0.05, **: 0.001 ≤ *p* < 0.01, ***: *p* ≤ 0.001.

At the species level, the top five species with the highest mean differences in relative read abundance across four seasons were *Phragmites australis*, *Lactuca indica*, *Euonymus japonicus*, *Bolboschoenus planiculmis*, and *Suaeda salsa* (Figure 7). Specifically, the proportion of *Phragmites australis* in spring was significantly higher than that in other three seasons; the proportion of *Lactuca indica* in summer was significantly higher than that in other three seasons, and the proportion of *Bolboschoenus planiculmis* in summer was significantly higher than that in both winter and spring; the proportion of *Euonymus japonicus* in winter was significantly higher than that in other three seasons; and the proportion of *Suaeda salsa* in autumn was significantly higher than that in spring (Figure 8).

## 4. Discussion

The availability of nutrients and plant resources significantly impacts the food composition of herbivores [31]. As a large mixed-feeding ungulate, the dietary composition of Milu essentially reflects the dynamic changes in available biomass, palatability, and nutritional quality of different plant species within its habitat across seasons. This mixed-feeding strategy, characterized by a morphology intermediate between grazing and browsing ruminants with a tendency towards bulk and roughage utilization, was early recognized based on the anatomy of the digestive system [32]. Investigating seasonal dietary variation helps to reveal how deer adjust their feeding strategies in response to fluctuations in environmental resources, thereby enhancing our understanding of their survival strategies in natural habitats [26].

Current studies have documented substantial regional differences in the annual diet range of Milu populations across different reserves. For instance, Wang et al. (2026) used DNA metabarcoding to reveal that Milu in Shishou National Nature Reserve consumed 283 plant species from 63 families and 31 orders throughout the year, while populations in Dachangshan National Nature Reserve, Dafeng National Nature Reserve, and Dongting Lake National Nature Reserves consumed 222, 228, and 99 plant species, respectively [27]. In the present study, we identified a total of 43 plant species belonging to 42 genera, 24 families, and 15 orders in the feces of Milu using high-throughput sequencing. Although the species count is relatively modest compared with other reserves, this reflects the localized vegetation composition of the Tianjin Qilihai Wetland yet demonstrates that Milu maintain a broad dietary spectrum consistent with their generalist herbivorous feeding habits [32]. The pronounced inter-population variation in dietary diversity underscores the importance of studying multiple populations to comprehensively understand the ecological flexibility and adaptive capacity of this species.

On an annual scale, *Phragmites australis* consistently remained one of the primary food sources across all seasons, a result consistent with studies from various geographic populations, including Yeyahu Wetland (Beijing), Dafeng Milu National Nature Reserve (Jiangsu), and our previous findings at the same study area [23,25,26,33,34,35]. This dietary consistency reflects two complementary ecological phenomena. First, it demonstrates a degree of dietary conservatism within the species [36,37], suggesting that Milu possess innate foraging preferences shaped by evolutionary history and physiological adaptation. Second, and equally importantly, it indicates that *Phragmites australis* is widely distributed across Chinese wetland ecosystems and possesses high availability across multiple habitat types [34]. Notably, differences in the staple plants consumed by Milu across studies are frequently attributable to variations in the local flora composition of different habitats. For example, some populations may emphasize *Robinia pseudoacacia* or native wetland forbs depending on regional vegetation structure. This pattern suggests that Milu possess strong behavioral flexibility, adapting their food selection to local conditions to sustain survival. This adaptive capacity is not merely a feeding response but rather a fundamental component of the species’ ecological niche breadth, demonstrating their strong adaptability to heterogeneous habitats and representing one of the key conditions for the successful reintroduction and population establishment across different geographic regions [26]. Furthermore, methodological differences among studies—such as the application of DNA metabarcoding versus traditional fecal microhistological analysis—can substantially impact the resolution and composition of detected dietary items [12], highlighting the need for methodological standardization when making historical comparisons or assessing long-term dietary changes.

In spring, *Phragmites australis* dominated the diet at an extraordinary proportion (72.79%), with Poaceae collectively accounting for 73.39%. Young reed tissues possess nutritional advantages particularly valuable during this period: low cellulose content [38], high moisture and crude protein, and excellent palatability and digestibility. These characteristics enable deer to rapidly restore winter-depleted body condition and accumulate breeding season reserves [38]. *Phragmites australis* begins extensive germination in early spring when most plants remain dormant, providing the most readily accessible, concentrated resource. This extreme dietary concentration reflects optimal foraging theory: when high-quality, abundant resources are available, herbivores minimize search costs by intensively exploiting the most profitable food source [39], making energy conservation through focused foraging metabolically rational.

Summer dietary composition shifted markedly, with *Lactuca indica*, *Bolboschoenus planiculmis*, and *Persicaria lapathifolia* becoming primary foods, as Asteraceae and Cyperaceae families collectively accounted for over 71%. Milu preferentially remain in or near water bodies during hot periods; this shift reflects both thermoregulatory behavior and critical changes in plant nutritional quality [26]. Grass stems rapidly become fibrous and lignified as summer progresses, increasing crude fiber and acid detergent fiber while protein and mineral contents decline [26,40]. Conversely, broadleaf herbaceous plants enter vigorous growth phases with leaves rich in crude protein and low in fiber, offering substantially higher nutritional value [41]. This nutritional trade-off drives the dietary transition to high-quality forbs. The selective preference for *Persicaria lapathifolia*, which contains condensed tannins, likely reflects phytochemical-mediated foraging preferences or functional responses to parasites and metabolic challenges [24]. It has been well documented that tannin-rich forage is not necessarily deleterious to ruminants; in fact, at appropriate concentrations, tannins can exert beneficial effects on protein metabolism and gastrointestinal health [24,42].

Autumn dietary composition exhibited the highest seasonal diversity, with top five families and species each representing less than 30% of the diet. This diversification reflects adaptive responses to declining autumn plant productivity and quality—many herbaceous species wither or lose nutritional value, forcing deer to expand their food portfolio to meet pre-winter energy demands [24]. Legume-dominated foraging increases markedly, particularly *Robinia pseudoacacia* and *Glycine soja*, as their high protein and lipid content facilitates critical fat accumulation before winter. However, *Glycine soja* represents a conservation concern: as a nationally protected plant (Category II), its heavy autumn consumption conflicts with rare plant protection objectives. Reserve management should establish long-term monitoring of *Glycine soja* populations, introduce high-protein alternative plants to redistribute foraging pressure, and implement spatial management to prevent localized overutilization while maintaining Milu food security.

The diet in winter has undergone significant changes, mainly relying on artificially cultivated plants such as *Euonymus japonicus* (31.06%), *Robinia pseudoacacia* (17.54%), and *Prunus cerasifera* (17.13%). This phenomenon indicates that during the winter period when natural food resources are scarce, artificial vegetation can serve as a crucial alternative food source, effectively alleviating seasonal nutritional constraints [43]. Particularly during ex situ conservation of endangered species and habitat reconstruction, planted vegetation not only provides a stable food supply but also enhances overwintering success of individual deer by extending effective foraging time and reducing energy expenditure. Therefore, in wetland conservation and management practices, scientifically planning and optimizing the structure of planted plant communities has undeniable positive significance for maintaining food security and habitat carrying capacity of threatened populations. Furthermore, despite their low nutritional value, the proportion of dried Poaceae plants in the winter diet increased compared to summer and autumn. This phenomenon may reflect their high availability and ease of access in the environment, serving as “filler food” to sustain basal metabolism and alleviate hunger when high-quality resources are scarce, reflecting the dietary broadening strategy of Milu under resource stress [44].

The seasonal differences in Milu diet are reflected not only in the diversity of plant taxa but also, more prominently, in the degree of concentration of major food resources and the underlying foraging strategy employed. The results reveal that deer adopt a highly specialized feeding strategy focused on a few plant species in spring and summer, whereas they shift to a more dispersed, broad-spectrum feeding strategy in autumn and winter [42]. From the data at both family and species levels, the diet in spring and summer shows striking concentration. In spring, Poaceae (73.39%) occupied an absolutely dominant position, within which *Phragmites australis* alone accounted for 72.79%. In summer, Asteraceae (47.42%) and Cyperaceae (24.42%) together accounted for over 71%, with *Lactuca indica* alone contributing 47.00% of the diet. This single dominant species pattern suggests that during the warm season when plant biomass and plant diversity are both at peak levels, the deer can efficiently forage on high-biomass, highly accessible, high-quality resources, conforming to optimal foraging theory’s predictions regarding selective utilization of energy-maximizing foods [39]. This specialized strategy may be related to the higher nutritional content (e.g., crude protein), superior palatability, and high abundance of these preferred plants during their growth season. The concentration of dietary effort on a small number of plant species during resource abundance represents an optimal solution to the classic foraging problem: when preferred foods are sufficiently abundant to meet all nutritional requirements, the metabolic cost of searching for and consuming alternative foods exceeds their nutritional benefit. Thus, specialization becomes energetically rational and behaviorally efficient.

In autumn and winter, dietary diversification becomes pronounced, with top five families and species each representing less than 30% of intake, reflecting fundamental environmental constraint shifts. As these seasons approach, dominant summer herbaceous plants wither or lose nutritional value, creating dual scarcity in both quantity and quality. Forced to expand food choices, deer increasingly consume woody plant stems, leaves, and fruits alongside residual herbs and seeds. This dietary broadening adheres to optimal foraging theory predictions: as preferred food abundance and quality decline, alternative foods become economically justifiable dietary additions even at lower profitability thresholds [39]. This seasonal strategy shift is not unique to Milu; European roe deer (*Capreolus capreolus*) similarly exhibit marked seasonality, feeding primarily on evergreen and herbaceous plants in autumn-winter but shifting to herbaceous plants and tree shoots in spring-summer [45]. Such seasonal foraging flexibility represents a general adaptive strategy among temperate cervids navigating predictable resource seasonality, reflecting deep evolutionary adaptation to temperate ecosystem dynamics.

Several plant species identified in this study have not been previously reported in Milu dietary studies from other regions, including *Lactuca indica*, *Euonymus japonicus*, *Rhus typhina*, and *Nelumbo nucifera*. And among them, *Lactuca indica* and *Euonymus japonicus* accounted for a high proportion in the results of this study. While some regional differences likely reflect genuine biogeographic plant community variation, methodological differences provide a more critical explanation. High-throughput sequencing offers substantially higher taxonomic resolution and sensitivity than traditional fecal microhistological analysis or stable isotope approaches, detecting trace amounts of easily digestible or fragmented food residue DNA [15,46]. These previously undetected plants likely represent historical dietary items undetected by traditional methods rather than recently adopted foods, highlighting an important methodological caveat: apparent dietary differences between studies may reflect analytical sensitivity differences rather than genuine biological variation in consumption patterns [12]. This underscores the need for methodological standardization in comparative dietary assessments.

This study also identified several plant species that have not been reported in dietary studies of Milu from other regions, such as *Lactuca indica*, *Euonymus japonicus*, *Rhus typhina*, and *Nelumbo nucifera*. Some of these differences may arise from variations in the vegetation types among different study areas, but a more important reason likely lies in methodological differences. Compared with traditional dietary analysis methods, high-throughput sequencing technology offers higher taxonomic resolution and sensitivity, enabling the detection of trace amounts of easily digestible or fragmented food residue DNA [15,46]. Therefore, this method may reveal plant taxa that were difficult to identify or easily overlooked by past morphological identification. This implies that these plants may not have been uneaten; rather, they might not have been effectively detected by some traditional methods. We acknowledge that the use of DNA based individual identification would further strengthen the assurance of independent sampling. However, given the conservation status of the Milu and the technical challenges of fecal DNA genotyping under wetland field conditions, we relied on a combination of spatial, morphological, and observational criteria to distinguish individuals. While not as precise as genetic markers, this approach is well established in ungulate dietary studies and, given the small population size (approximately 30 individuals) and high sampling intensity, provides a reliable basis for population level inference.

Overall, Milu exhibit high seasonal dietary plasticity, shifting from selective exploitation of few high-value plants during resource-abundant seasons (spring-summer) to opportunistic broad-spectrum foraging during resource-limited seasons (autumn-winter). This adaptive flexibility demonstrates the sophistication of their feeding ecology. The availability of nutrients and plant resources significantly impacts the food composition of herbivores. As a large mixed-feeding ungulate, the dietary composition of Milu essentially reflects the dynamic changes in available biomass, palatability, and nutritional quality of different plant species within its habitat across seasons. This mixed-feeding strategy, characterized by a morphology intermediate between grazing and browsing ruminants with a tendency towards bulk and roughage utilization, was early recognized based on the anatomy of the digestive system. Investigating sea-sonal dietary variation helps to reveal how deer adjust their feeding strategies in response to fluctuations in environmental resources, thereby enhancing our under-standing of their survival strategies in natural habitats. This study employed single rbcL markers with limited discriminatory power for closely related species [47,48]; future work should supplement with faster-evolving barcode regions such as trnL, increase seasonal sample sizes and implement multi-year continuous sampling spanning more annual cycles to comprehensively evaluate seasonal and inter-annual dietary variation and its demographic consequences in this critically endangered population.

## 5. Conclusions

This study demonstrates that the dietary composition of Milu in the Tianjin Qilihai Wetland undergoes pronounced seasonal shifts. In spring and summer, the deer adopt a highly specialized foraging strategy, intensively consuming high-quality, abundant resources such as *Phragmites australis* and *Lactuca indica*, a pattern consistent with optimal foraging theory. In autumn and winter, they transition to a broad-spectrum diet that includes woody plants, legumes, and cultivated species, reflecting adaptive responses to resource scarcity. *Phragmites australis* remains a year-round staple, owing both to dietary conservatism and to the widespread availability of this wetland dominant. However, the heavy autumn consumption of the nationally protected *Glycine soja* underscores the urgent need for population monitoring and the provisioning of alternative forages. The detection of previously unreported taxa—including *Lactuca indica* and *Euonymus japonicus*—via high-throughput sequencing highlights the substantial influence of methodological resolution on dietary assessments. Overall, Milu exhibit remarkable dietary plasticity, a key ecological trait that underpins their successful reintroduction and adaptation across diverse regions. Future research should integrate additional barcode markers and long-term sampling to better elucidate seasonal and inter-annual dietary dynamics and their demographic consequences, thereby providing a robust scientific basis for wetland conservation and adaptive management.

## Figures and Tables

**Table 1:** Sample grouping information.

Sampling Group	Sampling Time	Sample Size	Sample Number
Summer group	July 2024	8	Sum01–Sum08
Autumn group	October 2024	8	Aut01–Aut08
Winnter group	December 2024	7	Win01–Win07
Spring group	March 2025	11	Spr01–Spr011

## Data Availability

DNA sequencing data were deposited in the NCBI Sequence Read Archive (SRA) database under PRJNA1472920.

## Supplementary Materials

The following supporting information can be downloaded at https://www.mdpi.com/article/10.3390/ani16162544/s1: Figure S1: Rarefaction curves for all samples; Table S1: Proportion of plants in different seasons at species level; Table S2: Proportion of plants in different seasons at family level; Table S3: The information of all samples; Table S4: Alpha diversity index is based on *t*-test; Table S5: OTU Taxon Analysis.
