# Supplementary material for: Seasonal Diet Variation in Reintroduced Milu (Elaphurus davidianus) Revealed by DNA Macrobarcoding: Implications for Biodiversity Conservation
Source: Animals (Basel). 2026 Aug 14;16(16):2544. doi: 10.3390/ani16162544 (PMC13508985; doi:10.3390/ani16162544)
Supplement: Supplementary file 1 [file animals-16-02544-s001.zip › animals-4415821-supplementary.pdf]

Table S1 Proportion of plants in different seasons at species level

Plant species	Spring(%)	Summer(%)	Autumn(%)	Winter(%)
Phragmites australis	72.79%	3.49%	4.97%	15.18%
Lactuca indica	1.49%	47.00%	11.98%	1.07%
Robinia pseudoacacia	14.55%	0.39%	18.09%	17.54%
Euonymus japonicus	0.05%		0.00%	31.06%
Bolboschoenus planiculmis	0.08%	24.42%	4.93%	0.28%
Suaeda salsa	0.02%	1.77%	24.89%	0.03%
Prunus cerasifera	0.26%	0.20%	3.88%	17.13%
Persicaria lapathifolia		13.08%	0.00%	
Bassia scoparia		1.64%	3.69%	3.21%
Glycine soja	0.01%		9.49%	0.00%
Typha angustifolia	3.23%	4.00%	0.81%	0.19%
Ipomoea hederacea	0.19%	0.02%	2.33%	4.80%
Cynanchum chinense	0.39%	0.20%	4.64%	0.10%
Melica scabrosa	0.58%		4.08%	0.11%
Ulmus pumila	1.13%	0.13%	1.02%	2.04%
Rhus typhina		0.83%	2.12%	0.08%
Chenopodium album		0.15%	0.49%	1.80%
Rumex crispus	2.36%	0.00%	0.01%	
Ipomoea purpurea		0.00%	1.19%	0.91%
Amarantus retroflexus		0.00%	0.04%	
Salix babylonica	0.66%		0.00%	1.58%
Ipomoea purpurea	0.06%	0.00%	1.19%	0.91%
Amaranthus retroflexus	0.00%	0.00%	0.04%	1.96%
Styphnolobium japonicum	1.38%		0.01%	0.06%
Elymus dahuricus	0.02%	1.01%	0.00%	0.05%
Sonchus wightianus	0.01%	0.40%	0.53%	0.03%
Abutilon theophrasti		0.73%		
Polygonum aviculare		0.46%		
-FPOVSVT KBQPOJDVT			0.46%	
Cirsium arvense	0.02%	0.02%	0.21%	0.08%
Rosa multiflora	0.25%			0.01%
Melilotus officinalis	0.00%		0.07%	0.19%
Rubia cordifolia	0.18%			
Nelumbo nucifera	0.11%	0.00%	0.00%	0.06%
Bidens pilosa	0.01%		0.01%	0.15%
Setaria viridis	0.00%	0.00%	0.01%	0.12%

Lonicera maackii				0.09%
Ailanthus altissima		0.02%		0.07%
Fraxinus chinensis	0.07%	0.00%		
Lepidium apetalum	0.06%			
Humulus scandens	0.00%		0.02%	0.02%
**unclassified_f_Fabaceae**	0.02%		0.01%	
**unclassified_g_Aster**		0.00%	0.02%	0.01%
Artemisia annua	0.00%	0.00%	0.00%	

Table S2 Proportion of plants in different seasons at family level

Plant family	Spring(%)	Summer(%)	Autumn(%)	Winter(%)
**Poaceae**	73.39%	4.53%	9.06%	15.45%
**Asteraceae**	1.53%	47.42%	12.74%	1. 34%
**Fabaceae**	15.97%	0.39%	27.67%	17.79%
**Amaranthaceae**	0.03%	3.56%	29.12%	7.00%
**Celastraceae**	0.05%		0.00%	31.06%
**Cyperaceae**	0.08%	24.42%	4.93%	0.28%
**Rosaceae**	0.51%	0.20%	3.88%	17.13%
**Polygonaceae**	2.36%	13.55%	0.01%	
**Convolvulaceae**	0.26%	0.02%	3.52%	5.71%
**Typhaceae**	3.23%	4.00%	0.81%	0.19%
**Apocynaceae**	0.39%	0.20%	4.64%	0.10%
**Ulmaceae**	1.13%	0.13%	1.02%	2.04%
**Salicaceae**	0.66%		0.00%	1.58%
**Rubiaceae**	0.18%			
**Anacardiaceae**		0.83%	2.12%	0.08%
**Malvaceae**		0.73%		
**Lamiaceae**			0.46%	
**Nelumbonaceae**	0.11%	0.00%	0.00%	0.06%
**Caprifoliaceae**				0.09%
**Simaroubaceae**		0.02%		0.07%
**Oleaceae**	0.07%	0.00%		
**Tamaricaceae**		0.00%		
**Brassicaceae**	0.06%			
**Cannabaceae**	0.00%		0.02%	0.02%

Table S3 The information of all samples

SampleInfo	Seq_num	Base_num	Mean_length	Min_length	Max_length	OTU_num	Sequences
**Spr01**	26360	5323515	201.95429	200	203	26	23938
**Spr02**	109785	22171188	201.95098	200	203	10	23938
**Spr03**	28063	5667322	201.94997	200	203	7	23938
**Spr04**	25808	5212460	201.97071	200	203	19	23938
**Spr05**	24875	5023615	201.95437	200	203	10	23938
**Spr06**	28808	5817878	201.95356	200	203	23	23938
**Spr07**	24061	4859277	201.95657	200	203	19	23938
**Spr08**	33993	6865294	201.96199	200	203	25	23938
**Spr09**	32951	6654662	201.9563	200	203	4	23938
**Spr10**	25466	5143058	201.95783	200	203	12	23938
**Spr11**	24236	4894705	201.9601	200	203	12	23938
**Sum01**	54257	10956683	201.94045	200	203	18	23938
**Sum02**	45305	9148710	201.93599	200	225	19	23938
**Sum03**	42501	8582451	201.93527	200	203	16	23938
**Sum04**	60305	12177550	201.93268	200	203	21	23938
**Sum05**	50456	10189257	201.94342	200	203	22	23938
**Sum06**	34825	7035482	202.02389	200	472	13	23938
**Sum07**	61959	12512181	201.94291	200	206	15	23938
**Sum08**	60811	12280662	201.94804	200	210	21	23938
**Aut01**	91525	18482938	201.94415	200	252	15	23938
**Aut02**	65532	13234033	201.94764	200	235	26	23938
**Aut03**	55852	11278983	201.94412	200	203	15	23938
**Aut04**	61456	12409954	201.93234	200	203	30	23938
**Aut05**	62207	12561706	201.93396	200	240	32	23938
**Aut06**	52851	10672758	201.94051	200	235	22	23938
**Aut07**	50375	10173079	201.94698	200	203	21	23938
**Aut08**	61678	12456081	201.95339	200	250	33	23938
**Win01**	88097	17792519	201.9651	200	203	27	23938
**Win02**	65367	13200274	201.94095	200	204	32	23938
**Win03**	50869	10271301	201.91671	200	206	6	23938
**Win04**	59121	11941835	201.98973	200	251	25	23938
**Win05**	59989	12132352	202.24295	200	244	17	23938
**Win06**	63621	12850001	201.97735	200	238	14	23938
**Win07**	50960	10289737	201.91792	200	203	24	23938

Table S4 Alpha diversity index is based on t-test

Estimators	Autumn group-Mean	Autumn group-Sd	Spring group-Mean	Spring group-Sd	Summer group-Mean	Summer group-Sd	Winter group-Mean	Winter group-Sd	*P*_value	*P*_adjust	Statistic
shannon	1.4796	0.66694	0.80541	0.45967	1.1592	0.3992	1.3145	0.67976	0.05229	0.0732	7.71489
simpson	0.38569	0.25128	0.63864	0.21678	0.4835	0.19062	0.4234	0.29878	0.04689	0.0732	7.95794
ace	32.862	7.7791	15.603	10.736	22.13	5.6235	23.864	12.969	0.0064	0.0224	12.3082
Chao1	27.448	7.778	15.818	8.0165	20.438	5.2945	21.81	9.7982	0.0509	0.0732	7.77499
Pielou	0.46216	0.19361	0.29509	0.12262	0.39803	0.12074	0.42425	0.2087	0.1084	0.1084	6.06615

Table S5 OTU Taxon Analysis

domain	kingdom	phylum	class	order	family	genus	species	otu	Spr01	Spr02	Spr03	Spr04	Spr05	Spr06	Spr07	Spr08	Spr09	Spr10	Spr11	Sum01	Sum02	Sum03	Sum04	Sum05	Sum06	Sum07	Sum08	Aut01	Aut02	Aut03	Aut04	Aut05	Aut06	Aut07	Aut08	Win01	Win02	Win03	Win04	Win05	Win06	Win07	Total
d__Eukaryota	k__Viridiplantae	p__Tracheophyta	c__Magnoliopsida	o__Asterales	f__Asteraceae	g__*Artemisia*	s__*Artemisia_annua*	OTU85	0	6	0	0	0	0	0	0	0	0	0	2	0	0	0	1	0	0	4	0	1	0	1	0	0	0	1	0	0	0	0	0	0	0	16
d__Eukaryota	k__Viridiplantae	p__Tracheophyta	c__Magnoliopsida	o__Asterales	f__Asteraceae	g__*Aster*	s__unclassified_g__*Aster*	OTU93	0	0	0	0	0	0	0	0	0	0	0	1	2	0	0	1	0	0	0	0	0	0	0	41	0	0	0	0	0	0	0	0	11	1	57
d__Eukaryota	k__Viridiplantae	p__Tracheophyta	c__Magnoliopsida	o__Asterales	f__Asteraceae	g__*Bidens*	s__*Bidens_pilosa*	OTU57	0	0	0	0	0	0	0	19	0	0	0	0	0	0	0	0	0	0	0	0	0	0	0	2	0	0	0	0	47	0	20	0	0	172	260
d__Eukaryota	k__Viridiplantae	p__Tracheophyta	c__Magnoliopsida	o__Asterales	f__Asteraceae	g__*Bidens*	s__*Bidens_pilosa*	OTU59	0	0	0	0	0	0	0	0	0	0	0	0	0	0	0	0	0	0	0	0	4	0	3	3	0	0	0	2	1	0	2	0	0	3	18

domain	kingdom	phylum	class	order	family	genus	species	otu	Spr01	Spr02	Spr03	Spr04	Spr05	Spr06	Spr07	Spr08	Spr09	Spr10	Spr11	Sum01	Sum02	Sum03	Sum04	Sum05	Sum06	Sum07	Sum08	Aut01	Aut02	Aut03	Aut04	Aut05	Aut06	Aut07	Aut08	Win01	Win02	Win03	Win04	Win05	Win06	Win07	Total
d__Eukaryota	k__Viridiplantae	p__Tracheophyta	c__Magnoliopsida	o__Asterales	f__Asteraceae	g__*Cirsium*	s__*Cirsium_arvense*	OTU119	3	8	10	0	8	0	0	1	0	4	0	0	0	0	5	1	7	7	7	0	85	1	116	133	1	1	52	18	16	0	81	0	0	20	585
d__Eukaryota	k__Viridiplantae	p__Tracheophyta	c__Magnoliopsida	o__Asterales	f__Asteraceae	g__*Cirsium*	s__*Cirsium_arvense*	OTU29	1	2	5	0	2	0	0	0	0	2	0	0	1	0	0	3	2	1	0	0	5	0	1	1	0	0	1	0	0	0	1	0	0	0	28
d__Eukaryota	k__Viridiplantae	p__Tracheophyta	c__Magnoliopsida	o__Asterales	f__Asteraceae	g__*Lactuca*	s__*Lactuca_indica*	OTU31	406	290	276	0	815	0	454	18	0	1430	235	15551	20493	19735	8718	15402	395	235	9479	4205	4976	286	2919	7633	96	379	2454	368	636	0	664	0	0	126	118674
d__Eukaryota	k__Viridiplantae	p__Tracheophyta	c__Magnoliopsida	o__Asterales	f__Asteraceae	g__*Sonchus*	s__*Sonchus_wightianus*	OTU83	17	0	0	0	0	0	0	0	0	0	0	86	159	119	111	62	7	17	94	0	227	14	162	339	0	11	60	0	16	0	22	0	0	7	1530
d__Eukaryota	k__Viridiplantae	p__Tracheophyta	c__Magnoliopsida	o__Asterales	f__Asteraceae	g__*Sonchus*	s__*Sonchus_wightianus*	OTU90	0	0	0	3	1	0	0	0	0	0	0	8	9	62	3	6	0	3	5	0	52	24	7	28	11	23	17	0	1	0	5	0	0	1	269
d__Eukaryota	k__Viridiplantae	p__Tracheophyta	c__Magnoliopsida	o__Asterales	f__Asteraceae	g__*Sonchus*	s__*Sonchus_wightianus*	OTU95	0	0	0	0	0	0	0	0	0	0	0	1	6	0	1	4	0	0	3	0	7	1	7	11	0	0	5	0	1	0	0	0	0	0	47
d__Eukaryota	k__Viridiplantae	p__Tracheophyta	c__Magnoliopsida	o__Brassicales	f__Brassicaceae	g__*Lepidium*	s__*Lepidium_apetalum*	OTU8	151	0	0	0	0	0	0	0	0	0	0	0	0	0	0	0	0	0	0	0	0	0	0	0	0	0	0	0	0	0	0	0	0	0	151
d__Eukaryota	k__Viridiplantae	p__Tracheophyta	c__Magnoliopsida	o__Caryophyllales	f__Amaranthaceae	g__*Amaranthus*	s__*Amaranthus_retroflexus*	OTU117	0	0	0	0	0	0	0	0	0	0	0	0	0	0	0	0	0	0	0	0	0	0	0	0	0	0	0	0	0	0	44	0	0	0	44

domain	kingdom	phylum	class	order	family	genus	species	otu	Spr01	Spr02	Spr03	Spr04	Spr05	Spr06	Spr07	Spr08	Spr09	Spr10	Spr11	Sum01	Sum02	Sum03	Sum04	Sum05	Sum06	Sum07	Sum08	Aut01	Aut02	Aut03	Aut04	Aut05	Aut06	Aut07	Aut08	Win01	Win02	Win03	Win04	Win05	Win06	Win07	Total
d__Eukaryota	k__Viridiplantae	p__Tracheophyta	c__Magnoliopsida	o__Caryophyllales	f__Amaranthaceae	g__*Amaranthus*	s__*Amaranthus_retroflexus*	OTU94	0	0	0	3	0	0	0	0	0	0	0	0	0	0	0	0	0	0	2	0	0	0	0	79	0	5	0	0	0	0	3236	0	0	0	33325
d__Eukaryota	k__Viridiplantae	p__Tracheophyta	c__Magnoliopsida	o__Caryophyllales	f__Amaranthaceae	g__*Bassia*	s__*Bassia_scoparia*	OTU42	0	0	0	0	0	0	0	0	0	0	0	426	0	0	0	1355	0	1032	326	5137	702	69	101	27	581	39	410	536	56	0	0	4786	0	1	155844
d__Eukaryota	k__Viridiplantae	p__Tracheophyta	c__Magnoliopsida	o__Caryophyllales	f__Amaranthaceae	g__*Chenopodium*	s__*Chenopodium_album*	OTU34	0	0	0	0	0	0	0	0	0	0	0	0	196	0	0	0	0	93	0	0	0	0	586	0	0	83	276	526	13	0	2302	0	0	177	42252
d__Eukaryota	k__Viridiplantae	p__Tracheophyta	c__Magnoliopsida	o__Caryophyllales	f__Amaranthaceae	g__*Suaeda*	s__*Suaeda_salsa*	OTU153	0	0	0	0	0	0	0	0	0	0	0	0	0	74	0	0	2	30	0	0	3	5	0	0	15	9	2	0	0	0	0	0	0	0	140
d__Eukaryota	k__Viridiplantae	p__Tracheophyta	c__Magnoliopsida	o__Caryophyllales	f__Amaranthaceae	g__*Suaeda*	s__*Suaeda_salsa*	OTU50	0	0	0	0	0	0	0	0	0	0	0	5	0	124	0	15	6	12	1	1	11	1	0	0	1	6	3	0	0	0	0	0	0	1	187
d__Eukaryota	k__Viridiplantae	p__Tracheophyta	c__Magnoliopsida	o__Caryophyllales	f__Amaranthaceae	g__*Suaeda*	s__*Suaeda_salsa*	OTU52	0	0	0	65	0	0	0	0	0	0	0	0	0	1696	0	0	477	941	1	1762	2598	20774	318	848	293	19695	1326	2	20	0	23	5	0	5	508492
d__Eukaryota	k__Viridiplantae	p__Tracheophyta	c__Magnoliopsida	o__Caryophyllales	f__Polygonaceae	g__*Persicaria*	s__*Persicaria_lapathifolia*	OTU62	0	0	0	0	0	0	0	0	0	0	0	1294	501	13	6280	2017	3568	7356	4024	0	0	0	0	0	0	1	0	0	0	0	0	0	0	0	250554

domain	kingdom	phylum	class	order	family	genus	species	otu	Spr01	Spr02	Spr03	Spr04	Spr05	Spr06	Spr07	Spr08	Spr09	Spr10	Spr11	Sum01	Sum02	Sum03	Sum04	Sum05	Sum06	Sum07	Sum08	Aut01	Aut02	Aut03	Aut04	Aut05	Aut06	Aut07	Aut08	Win01	Win02	Win03	Win04	Win05	Win06	Win07	Total
d__Eukaryota	k__Viridiplantae	p__Tracheophyta	c__Magnoliopsida	o__Caryophyllales	f__Polygonaceae	g__*Polygonum*	s__*Polygonum_aviculare*	OTU61	0	0	0	0	0	0	0	0	0	0	0	291	0	0	314	0	0	0	280	0	0	0	0	0	0	0	0	0	0	0	0	0	0	0	885
d__Eukaryota	k__Viridiplantae	p__Tracheophyta	c__Magnoliopsida	o__Caryophyllales	f__Polygonaceae	g__*Rumex*	s__*Rumex_crispus*	OTU6	411	0	0	1838	0	2289	1461	203	0	0	0	0	0	0	4	0	0	0	1	20	0	0	0	0	0	0	0	0	0	0	0	0	0	0	6227
d__Eukaryota	k__Viridiplantae	p__Tracheophyta	c__Magnoliopsida	o__Caryophyllales	f__Tamaricaceae	g__*Tamarix*	s__*Tamarix_chinensis*	OTU75	0	0	0	0	0	0	0	0	0	0	0	0	0	0	0	0	3	0	0	0	0	0	0	0	0	0	0	0	0	0	0	0	0	0	3
d__Eukaryota	k__Viridiplantae	p__Tracheophyta	c__Magnoliopsida	o__Celastrales	f__Celastraceae	g__*Euonymus*	s__*Euonymus_japonicus*	OTU110	0	0	0	0	0	0	0	1	0	0	0	0	0	0	0	0	0	0	0	0	0	0	0	1	0	0	0	33	245	3	2	126	2	147	560
d__Eukaryota	k__Viridiplantae	p__Tracheophyta	c__Magnoliopsida	o__Celastrales	f__Celastraceae	g__*Euonymus*	s__*Euonymus_japonicus*	OTU22	0	0	0	0	0	118	0	0	0	0	0	0	0	0	0	0	0	0	0	0	0	0	0	0	1	0	0	10400	8296	23808	2	7218	0	1760	51603
d__Eukaryota	k__Viridiplantae	p__Tracheophyta	c__Magnoliopsida	o__Dipsacales	f__Caprifoliaceae	g__*Lonicera*	s__*Lonicera_maackii*	OTU108	0	0	0	0	0	0	0	0	0	0	0	0	0	0	0	0	0	0	0	0	0	0	0	0	0	0	0	108	0	0	0	0	0	49	157
d__Eukaryota	k__Viridiplantae	p__Tracheophyta	c__Magnoliopsida	o__Fabales	f__Fabaceae	g__*Glycine*	s__*Glycine_max*	OTU101	0	0	0	0	0	0	1	0	0	0	0	0	0	0	0	0	0	0	0	0	0	0	0	1	32	0	0	0	0	0	0	0	0	0	34

domain	kingdom	phylum	class	order	family	genus	species	otu	Spr01	Spr02	Spr03	Spr04	Spr05	Spr06	Spr07	Spr08	Spr09	Spr10	Spr11	Sum01	Sum02	Sum03	Sum04	Sum05	Sum06	Sum07	Sum08	Aut01	Aut02	Aut03	Aut04	Aut05	Aut06	Aut07	Aut08	Win01	Win02	Win03	Win04	Win05	Win06	Win07	Total
d__Eukaryota	k__Viridiplantae	p__Tracheophyta	c__Magnoliopsida	o__Fabales	f__Fabaceae	g__*Glycine*	s__*Glycine_max*	OTU103	2	14	0	6	0	6	1	9	0	0	0	0	0	0	0	0	0	0	0	0	0	0	1	1	18139	0	1	0	0	0	0	3	3	0	18186
d__Eukaryota	k__Viridiplantae	p__Tracheophyta	c__Magnoliopsida	o__Fabales	f__Fabaceae	g__*Melilotus*	s__*Melilotus_officinalis*	OTU104	1	0	0	0	0	0	0	0	0	0	0	0	0	0	0	0	0	0	0	0	0	0	0	0	0	0	127	312	0	0	2	2	0	0	444
d__Eukaryota	k__Viridiplantae	p__Tracheophyta	c__Magnoliopsida	o__Fabales	f__Fabaceae	g__*Robinia*	s__*Robinia_pseudoacacia*	OTU5	4330	1	0	6528	5	9125	4447	13876	0	0	1	277	0	1	314	0	0	0	149	793	6605	0	9703	7397	600	2	9549	6573	3366	0	3598	3718	11771	373	103102
d__Eukaryota	k__Viridiplantae	p__Tracheophyta	c__Magnoliopsida	o__Fabales	f__Fabaceae	g__*Styphnolobium*	s__*Styphnolobium_japonicum*	OTU126	231	0	1	421	1	534	283	878	0	0	0	0	0	0	0	0	0	0	0	0	0	0	1	1	9	0	4	0	16	0	0	69	0	0	2449
d__Eukaryota	k__Viridiplantae	p__Tracheophyta	c__Magnoliopsida	o__Fabales	f__Fabaceae	g__*Styphnolobium*	s__*Styphnolobium_japonicum*	OTU18	42	0	0	224	0	110	65	154	0	0	0	0	0	0	0	0	0	0	0	0	0	0	0	0	0	0	0	0	2	0	0	0	0	0	597
d__Eukaryota	k__Viridiplantae	p__Tracheophyta	c__Magnoliopsida	o__Fabales	f__Fabaceae	g__*Styphnolobium*	s__*Styphnolobium_japonicum*	OTU26	9	0	0	14	0	23	10	41	0	0	0	0	0	0	0	0	0	0	0	0	0	0	0	0	0	0	0	0	0	0	0	0	0	0	97
d__Eukaryota	k__Viridiplantae	p__Tracheophyta	c__Magnoliopsida	o__Fabales	f__Fabaceae	g__*Styphnolobium*	s__*Styphnolobium_japonicum*	OTU49	60	0	0	162	0	108	61	192	0	0	0	0	0	0	0	0	0	0	0	0	0	0	0	0	2	0	0	0	0	0	0	5	0	0	590
d__Eukaryota	k__Viridiplantae	p__Tracheophyta	c__Magnoliopsida	o__Fabales	f__Fabaceae	g__*Styphnolobium*	s__*Styphnolobium_japonicum*	OTU92	0	0	0	0	0	0	0	0	0	0	0	0	0	0	0	0	0	0	0	0	0	0	2	2	0	0	0	0	0	0	2	0	1	0	7

domain	kingdom	phylum	class	order	family	genus	species	otu	Spr01	Spr02	Spr03	Spr04	Spr05	Spr06	Spr07	Spr08	Spr09	Spr10	Spr11	Sum01	Sum02	Sum03	Sum04	Sum05	Sum06	Sum07	Sum08	Aut01	Aut02	Aut03	Aut04	Aut05	Aut06	Aut07	Aut08	Win01	Win02	Win03	Win04	Win05	Win06	Win07	Total
d__Eukaryota	k__Viridiplantae	p__Tracheophyta	c__Magnoliopsida	o__Fabales	f__Fabaceae	g__unclassified_f__Fabaceae	s__unclassified_f__Fabaceae	OTU97	8	1	0	4	0	6	2	43	0	0	0	0	0	0	0	0	0	0	0	0	1	0	0	0	11	0	1	0	0	0	0	0	0	0	77
d__Eukaryota	k__Viridiplantae	p__Tracheophyta	c__Magnoliopsida	o__Gentianales	f__Apocynaceae	g__*Cynanchum*	s__*Cynanchum_chinense*	OTU7	214	0	0	36	0	184	510	78	0	0	0	0	263	0	0	122	0	0	0	0	0	2540	366	193	2000	3457	326	84	77	0	0	0	0	0	10450
d__Eukaryota	k__Viridiplantae	p__Tracheophyta	c__Magnoliopsida	o__Gentianales	f__Rubiaceae	g__*Rubia*	s__*Rubia_cordifolia*	OTU25	0	0	0	0	0	0	0	52	0	0	435	0	0	0	0	0	0	0	0	0	0	0	0	0	0	0	0	0	0	0	0	0	0	0	487
d__Eukaryota	k__Viridiplantae	p__Tracheophyta	c__Magnoliopsida	o__Lamiales	f__Lamiaceae	g__*Leonurus*	s__*Leonurus_japonicus*	OTU86	0	0	0	0	0	0	0	0	0	0	0	0	0	0	0	0	0	0	0	872	0	0	0	0	0	0	0	0	0	0	0	0	0	0	872
d__Eukaryota	k__Viridiplantae	p__Tracheophyta	c__Magnoliopsida	o__Lamiales	f__Oleaceae	g__*Fraxinus*	s__*Fraxinus_chinensis*	OTU2	103	0	0	0	0	0	0	0	0	90	0	0	0	0	1	0	0	0	0	0	0	0	0	0	0	0	0	0	0	0	0	0	0	0	194
d__Eukaryota	k__Viridiplantae	p__Tracheophyta	c__Magnoliopsida	o__Malpighiales	f__Salicaceae	g__*Salix*	s__*Salix_babylonica*	OTU3	271	0	0	40	0	485	462	135	0	120	230	0	0	0	0	0	0	0	0	1	0	0	0	0	1	0	1	186	104	0	2057	308	0	0	4401
d__Eukaryota	k__Viridiplantae	p__Tracheophyta	c__Magnoliopsida	o__Malvales	f__Malvaceae	g__*Abutilon*	s__*Abutilon_theophrasti*	OTU66	0	0	0	0	0	0	0	0	0	0	0	0	0	109	188	1106	0	0	0	0	0	0	0	0	0	0	0	0	0	0	0	0	0	0	1403
d__Eukaryota	k__Viridiplantae	p__Tracheophyta	c__Magnoliopsida	o__Poales	f__Cyperaceae	g__*Bolboschoenus*	s__*Bolboschoenus_planiculmis*	OTU111	0	0	0	0	0	0	0	0	0	204	0	0	0	0	0	0	0	0	0	4	0	0	0	0	0	0	0	0	444	0	0	0	0	0	652

domain	kingdom	phylum	class	order	family	genus	species	otu	Spr01	Spr02	Spr03	Spr04	Spr05	Spr06	Spr07	Spr08	Spr09	Spr10	Spr11	Sum01	Sum02	Sum03	Sum04	Sum05	Sum06	Sum07	Sum08	Aut01	Aut02	Aut03	Aut04	Aut05	Aut06	Aut07	Aut08	Win01	Win02	Win03	Win04	Win05	Win06	Win07	Total
d__Eukaryota	k__Viridiplantae	p__Tracheophyta	c__Magnoliopsida	o__Poales	f__Cyperaceae	g__*Bolboschoenus*	s__*Bolboschoenus_planiculmis*	OTU39	0	0	0	0	0	0	0	0	0	0	0	0	0	0	0	0	1	0	0	0	0	0	0	0	0	0	0	0	20	0	0	0	0	0	21
d__Eukaryota	k__Viridiplantae	p__Tracheophyta	c__Magnoliopsida	o__Poales	f__Cyperaceae	g__*Bolboschoenus*	s__*Bolboschoenus_planiculmis*	OTU63	0	0	0	0	0	0	0	0	0	1	0	3833	352	23	3679	420	19022	13144	6298	8961	10	191	24	52	0	115	85	0	8	0	0	0	0	0	56218
d__Eukaryota	k__Viridiplantae	p__Tracheophyta	c__Magnoliopsida	o__Poales	f__Poaceae	g__*Elymus*	s__*Elymus_dahuricus*	OTU24	0	0	0	0	0	0	0	60	1	0	0	0	905	243	0	790	0	0	0	0	0	0	0	1	0	1	1	1	75	0	0	0	0	0	2078
d__Eukaryota	k__Viridiplantae	p__Tracheophyta	c__Magnoliopsida	o__Poales	f__Poaceae	g__*Melica*	s__*Melica_scabrosa*	OTU15	0	291	343	0	241	2	0	0	469	0	172	0	0	0	0	0	0	0	0	0	1669	0	1895	1789	0	0	2462	171	0	0	0	0	9	0	9513
d__Eukaryota	k__Viridiplantae	p__Tracheophyta	c__Magnoliopsida	o__Poales	f__Poaceae	g__*Phragmites*	s__*Phragmites_australis*	OTU4	16667	22231	22635	13774	22280	8862	1552	7415	21347	20653	20145	372	471	570	1940	1194	389	1017	728	1073	1418	0	2693	2319	806	41	1171	1104	1524	0	7938	7106	7463	398	233296
d__Eukaryota	k__Viridiplantae	p__Tracheophyta	c__Magnoliopsida	o__Poales	f__Poaceae	g__*Setaria*	s__*Setaria_viridis*	OTU139	0	0	0	0	0	2	0	4	0	0	0	0	1	2	14	37	0	0	3	0	0	0	2	5	1	0	4	0	0	0	1	0	197	0	273
d__Eukaryota	k__Viridiplantae	p__Tracheophyta	c__Magnoliopsida	o__Poales	f__Typhaceae	g__*Typha*	s__*Typha_angustifolia*	OTU1	600	1094	668	0	330	368	0	526	2121	1423	1377	1763	402	1163	1860	1004	59	49	1353	0	15	0	623	280	0	0	633	0	151	0	0	0	0	169	18031

domain	kingdom	phylum	class	order	family	genus	species	otu	Spr01	Spr02	Spr03	Spr04	Spr05	Spr06	Spr07	Spr08	Spr09	Spr10	Spr11	Sum01	Sum02	Sum03	Sum04	Sum05	Sum06	Sum07	Sum08	Aut01	Aut02	Aut03	Aut04	Aut05	Aut06	Aut07	Aut08	Win01	Win02	Win03	Win04	Win05	Win06	Win07	Total
d__Eukaryota	k__Viridiplantae	p__Tracheophyta	c__Magnoliopsida	o__Proteales	f__Nelumbonaceae	g__*Nelumbo*	s__*Nelumbo_nucifera*	OTU106	0	0	0	38	255	3	0	1	0	0	0	0	0	0	0	0	0	1	0	0	1	0	2	0	0	0	0	100	0	0	0	0	0	0	401
d__Eukaryota	k__Viridiplantae	p__Tracheophyta	c__Magnoliopsida	o__Rosales	f__Cannabaceae	g__*Humulus*	s__*Humulus_scandens*	OTU27	0	0	0	0	0	0	0	0	0	3	0	0	0	0	0	0	0	0	0	0	1	0	3	22	0	1	2	36	0	0	0	0	0	0	68
d__Eukaryota	k__Viridiplantae	p__Tracheophyta	c__Magnoliopsida	o__Rosales	f__Rosaceae	g__*Prunus*	s__*Prunus_cerasifera*	OTU107	4	0	0	0	0	1	22	7	0	0	0	0	5	0	0	16	0	0	0	0	0	0	0	0	0	0	0	34	274	5	0	10	0	98	476
d__Eukaryota	k__Viridiplantae	p__Tracheophyta	c__Magnoliopsida	o__Rosales	f__Rosaceae	g__*Prunus*	s__*Prunus_cerasifera*	OTU43	125	0	0	0	0	24	430	51	0	0	23	1	61	1	0	294	0	0	0	0	4322	1	305	79	0	0	2721	1183	7360	4	22	25	80	19602	36714
d__Eukaryota	k__Viridiplantae	p__Tracheophyta	c__Magnoliopsida	o__Rosales	f__Rosaceae	g__*Rosa*	s__*Rosa_multiflora*	OTU20	0	0	0	663	0	0	0	0	0	0	1	0	0	0	0	0	0	0	0	0	0	0	0	0	0	0	0	6	0	0	0	0	0	3	673
d__Eukaryota	k__Viridiplantae	p__Tracheophyta	c__Magnoliopsida	o__Rosales	f__Ulmaceae	g__*Ulmus*	s__*Ulmus_pumila*	OTU19	173	0	0	36	0	1017	21	106	0	5	1050	11	104	3	12	87	0	0	31	12	2	0	86	1796	0	0	56	80	24	96	287	460	1470	617	7642
d__Eukaryota	k__Viridiplantae	p__Tracheophyta	c__Magnoliopsida	o__Rosales	f__Ulmaceae	g__*Ulmus*	s__*Ulmus_pumila*	OTU33	48	0	0	7	0	201	6	56	0	3	258	0	0	0	0	1	0	0	0	0	0	0	3	0	0	0	2	59	13	22	69	85	87	51	971
d__Eukaryota	k__Viridiplantae	p__Tracheophyta	c__Magnoliopsida	o__Sapindales	f__Anacardiaceae	g__*Rhus*	s__*Rhus_typhina*	OTU69	0	0	0	0	0	0	0	0	0	0	0	0	0	0	460	0	0	0	1139	0	338	0	749	369	1136	47	1422	10	116	0	0	0	0	0	5786

domain	kingdom	phylum	class	order	family	genus	species	otu	Spr01	Spr02	Spr03	Spr04	Spr05	Spr06	Spr07	Spr08	Spr09	Spr10	Spr11	Sum01	Sum02	Sum03	Sum04	Sum05	Sum06	Sum07	Sum08	Aut01	Aut02	Aut03	Aut04	Aut05	Aut06	Aut07	Aut08	Win01	Win02	Win03	Win04	Win05	Win06	Win07	Total
d__Eukaryota	k__Viridiplantae	p__Tracheophyta	c__Magnoliopsida	o__Sapindales	f__Simaroubaceae	g__*Ailanthus*	s__*Ailanthus_altissima*	OTU73	0	0	0	0	0	0	0	0	0	0	0	0	0	0	29	0	0	0	0	0	0	0	0	0	0	0	0	0	119	0	0	0	0	0	148
d__Eukaryota	k__Viridiplantae	p__Tracheophyta	c__Magnoliopsida	o__Solanales	f__Convolvulaceae	g__*Ipomoea*	s__*Ipomoea_hederacea*	OTU159	4	0	0	0	0	7	0	0	0	0	0	1	0	0	1	0	0	0	0	5	20	2	45	19	6	1	4	22	22	0	73	0	60	6	298
d__Eukaryota	k__Viridiplantae	p__Tracheophyta	c__Magnoliopsida	o__Solanales	f__Convolvulaceae	g__*Ipomoea*	s__*Ipomoea_hederacea*	OTU21	54	0	0	76	0	298	49	12	0	0	11	15	3	0	3	0	0	0	10	1033	428	19	1950	318	194	13	405	2055	770	0	2148	11	2726	151	12752
d__Eukaryota	k__Viridiplantae	p__Tracheophyta	c__Magnoliopsida	o__Solanales	f__Convolvulaceae	g__*Ipomoea*	s__*Ipomoea_purpurea*	OTU35	3	0	0	0	0	165	1	0	0	0	0	0	4	0	1	0	0	0	0	59	437	10	1264	148	2	8	354	29	105	0	1337	1	58	0	3986

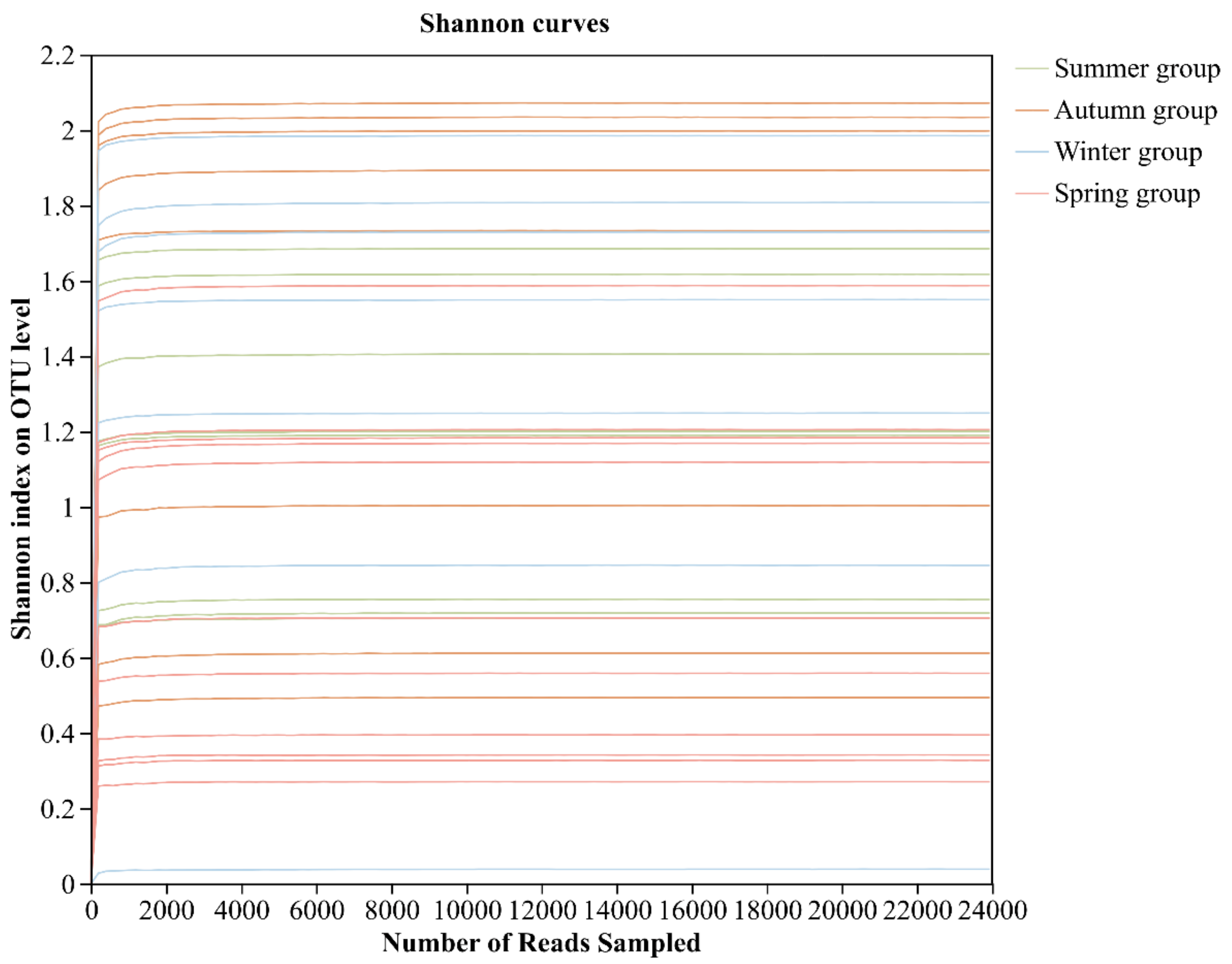

Figure S1 Rarefaction curves for all samples
